# Supplementary material for: The Kinetics of the Redox Reaction of Platinum(IV) Ions with Ascorbic Acid in the Presence of Oxygen
Source: Materials (Basel). 2023 Jun 27;16(13):4630. doi: 10.3390/ma16134630 (PMC10342784; doi:10.3390/ma16134630)
Supplement: Supplementary file 1 [file materials-16-04630-s001.zip › materials-2347065-supplementary.pdf]

Supplementary Materials

The Kinetics of the Redox Reaction of Platinum(IV) Ions with Ascorbic Acid in the Presence of Oxygen

Magdalena Luty-Błocho [1,*], Aleksandra Szot [1], Volker Hessel [2] and Krzysztof Fitzner [1]

[1] Faculty of Non-Ferrous Metals, AGH University of Krakow, al. Adama Mickiewicza 30, 30-059 Kraków, Poland; szot@agh.edu.pl (A.S.); fitzner@agh.edu.pl (K.F.)
[2] School of Chemical Engineering, The University of Adelaide, Adelaide 5005, Australia; volker.hessel@adelaide.edu.au
* Correspondence: mlb@agh.edu.pl; Tel.: +48-12-617-4126

S1. The spectra evolution in the course of the PtNPs formation.

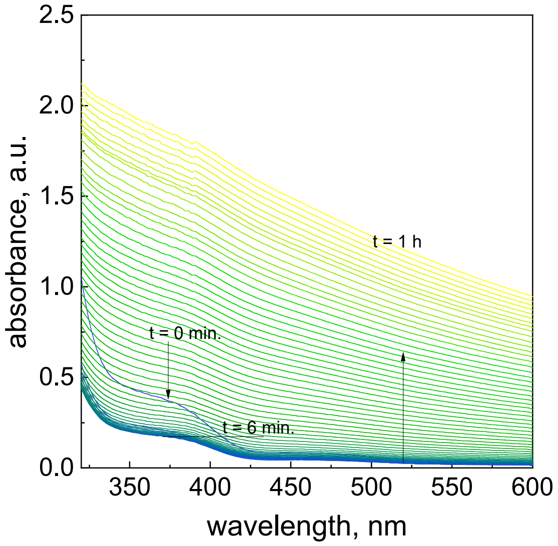

(**a**)

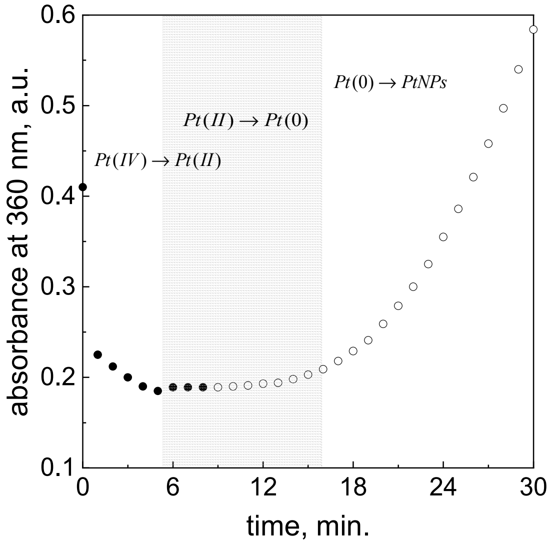

(**b**)

Figure S1. The change in the turbidity level characteristic for the process of platinum nanoparticles formation (a), kinetic curve registered at 360 nm (b). Conditions: $C_{0, Pt(IV)}$ = 1.0 mM,$C_{0, H2Asc}$ = 60 mM, pH = 2.50 ± 0.05, T = 313 ± 0.1 K, I = 0.06 M.

S2. The Arrhenius and Eyring dependency

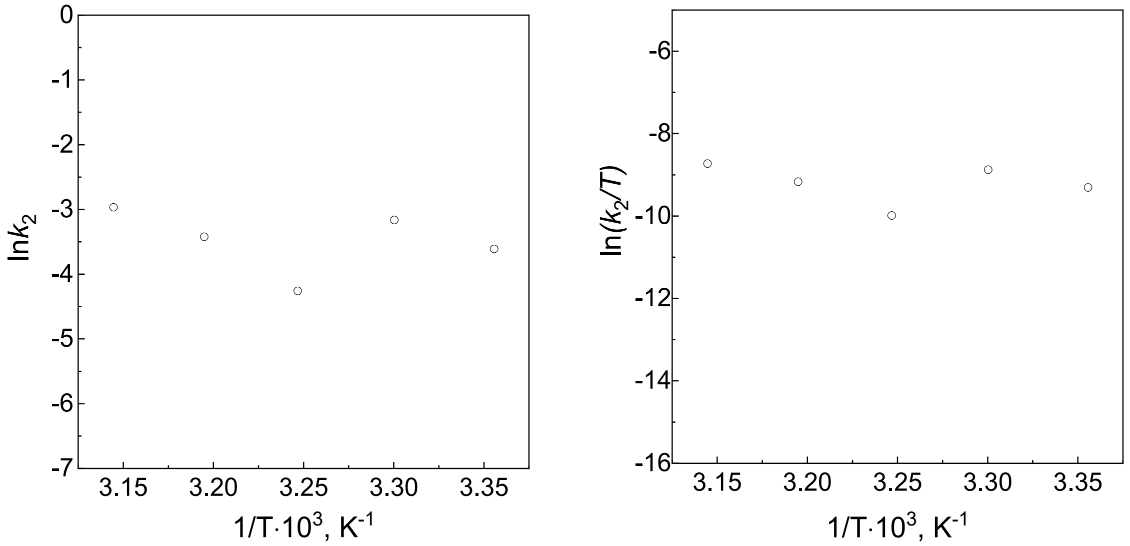

(**a**) (**b**)

Figure S2. Arrhenius (a) and Eyring (b) dependency for reduction of Pt(IV) ions using ascorbic acid. Conditions: $C_{0,Pt(IV)}$ = 1.0 mM, $C_{0,H2Asc}$ = 60 mM, pH = 2.50 ± 0.05, I = 0.06 M.

S3. The kinetic curves registered at different pH of the solution.

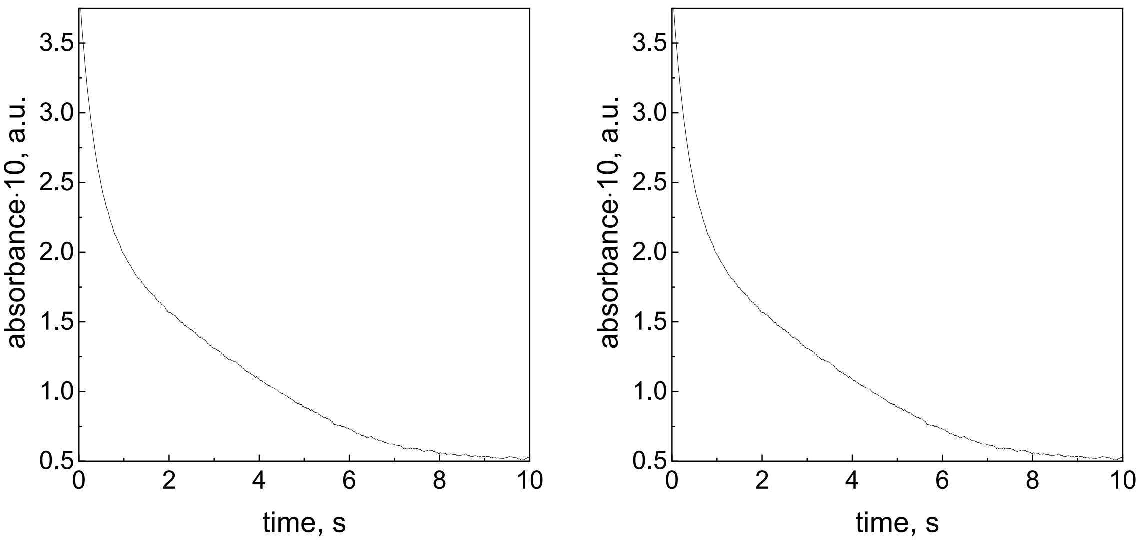

(**a**) (b)

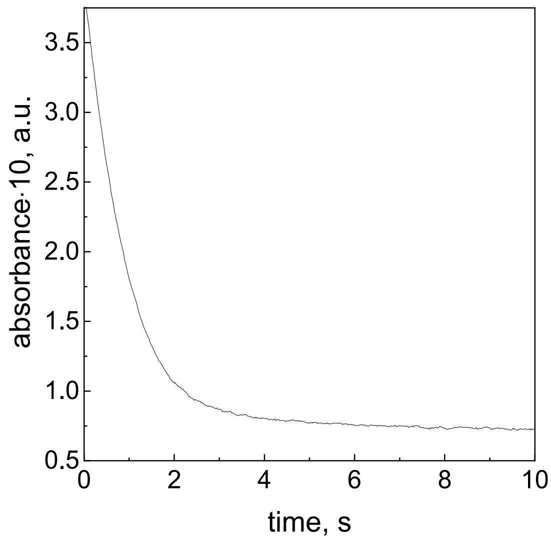

(**c**)

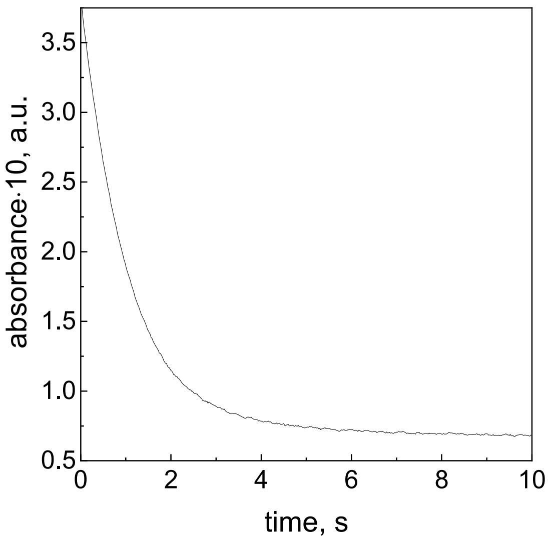

(**d**)

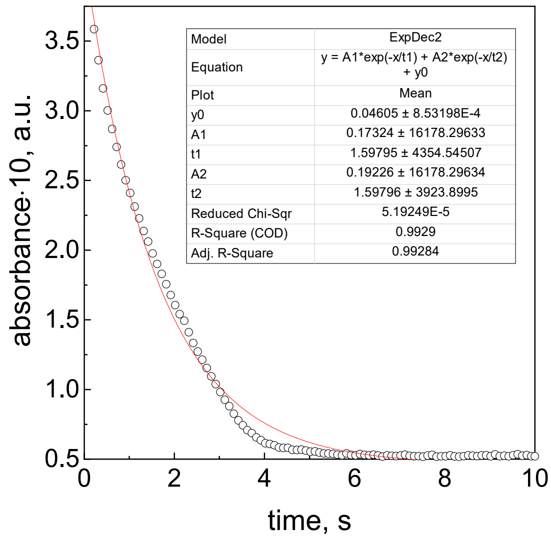

(**e**)

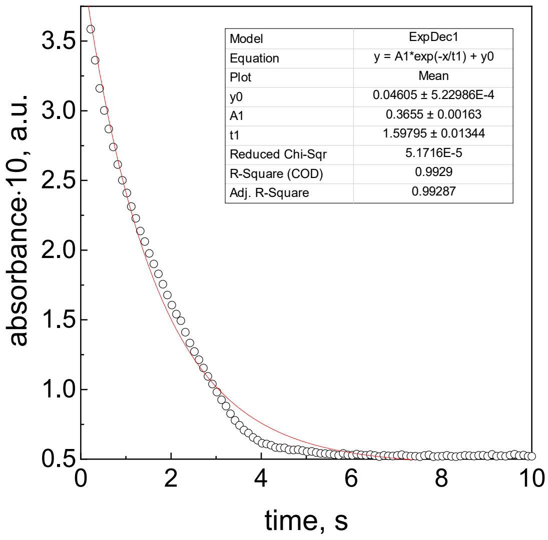

(**f**)

Figure S3. The sample of kinetic curves at different pH levels of the solution, registered during Pt(IV) ion reduction using ascorbic acid: pH = 2.15 (a), 3.65 (b), 4.17 (c) and 5.04 (d). The sample of kinetic curve with fitting curve (double exponential (e) and single exponential (f) for reaction carried out at pH = 3.65). Conditions: $C_{0,Pt(IV)}$ = 1.0 mM, $C_{0,\ H2Asc}$ = 60 mM, T = 313 ± 0.1 K, I = 0.06 M.

S4. Kinetic curves at different ionic strength

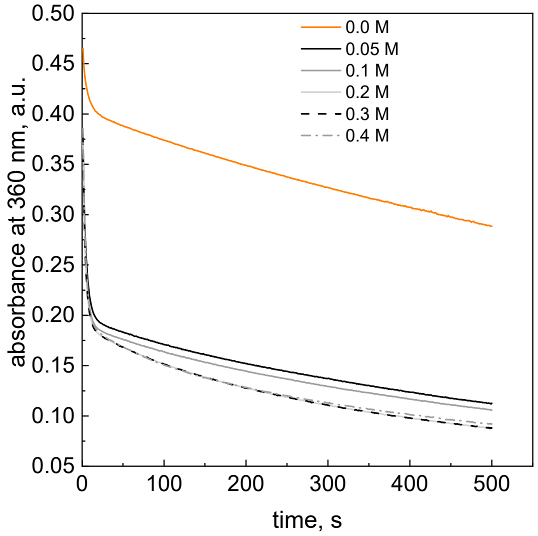

Figure S4. The sample of kinetic curve registered during Pt(IV) ion reduction using ascorbic acid. Conditions: $C_{0,Pt(IV)}$ = 1.0 mM, $C_{0,\ H2Asc}$ = 60 mM, T = 313 ± 0.1 K, pH = 2.5.

S5. The influence of chloride concentration on the share of platinum species

Table S1. The influence of chloride ions concentration on the share of the platinum species. Conditions: $C_{0,Pt(IV)}$ = 1.00 mM, $C_{0,H2Asc}$ = 60.0 mM, [Na$^+$] = 0.4 M, T = 313 ± 0.1 K, pH = 2.50 ± 0.5, I = 0.4 M.

[Cl$^-$] M	$y_0 \propto C_{[PtCl_4]^{2-}}$ a.u.	$A_1 \propto C_{[PtCl_6]^{2-}}$ a.u.	$A_2 \propto C_{[PtCl_5(H_2O)]^-}$ a.u.	$C_{[PtCl_4]^{2-}}$, mM	$C_{[PtCl_6]^{2-}}$, mM	$C_{[PtCl_5(H_2O)]^-}$, mM
0.0	0.067	0.18	0.14	1.12	0.367	0.285
0.05	0.025	0.14	0.19	0.446	0.286	0.388
0.10	0.03	0.2	0.16	0.536	0.408	0.326
0.20	0.03	0.25	0.15	0.536	0.510	0.306
0.30	0.04	0.26	0.135	0.714	0.531	0.276
0.40	0.05	0.27	0.13	0.893	0.551	0.265

$C_{[PtCl_6]^{2-}} = A_1/\varepsilon$, $C_{[PtCl_5(H_2O)]^-} = A_2/\varepsilon$, $C_{[PtCl_4]^{2-}} = y_0/\varepsilon_{Pt(II)}$, where ε = 490 M^{-1}cm^{-1}, $\varepsilon_{Pt(II)}$ = 56 M^{-1}cm^{-1}.

S6. The influence of the oxygen on kinetic curves.

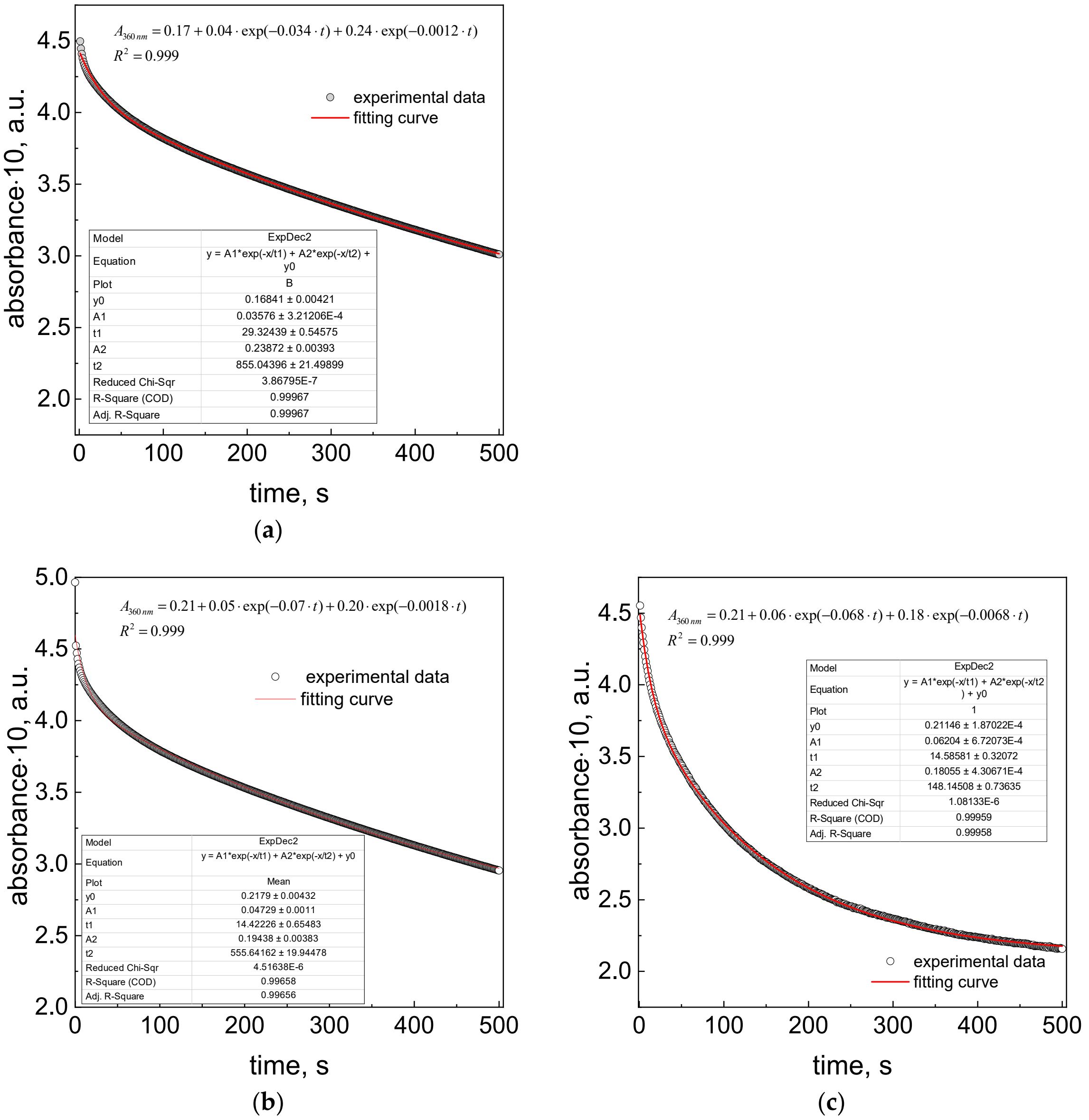

Figure S5. The sample of kinetic curve with fitting registered during Pt(IV) ion reduction using ascorbic acid containing oxygen (a,b) and in deaerated solution (c). Conditions: $C_{0,Pt(IV)}$ = 1.0 mM, $C_{0,\ H2Asc}$ = 60 mM (a), 2.0 mM (b, c), T = 313 ± 0.1 K, I = 0.06 M, pH = 2.5.

Table S2. Experimental and calculated values of observed rate constants.

**Solution**	Amount of reductant, mM	$k_{1,obs}$ s^{-1}	$k_{2,\ obs}$ s^{-1}	Degree of dissociation, α^1, %
Contains O_2	60 mM	0.034	0.0012	4
	2 mM	0.07	0.0018	20
Deaerated	2 mM	0.068	0.0068	20

[1] $\alpha, \% = \alpha \cdot 100\%$, where $\alpha = \sqrt{\frac{Ka}{c}}$, and Ka – dissociation constant for ascorbic acid ($K_a = 10^{-pK_a}$, $pK_a = 4.1$) and equals $7.9 \cdot 10^{-5}$.

S7. Sample of kinetic curves registered at different wavelength

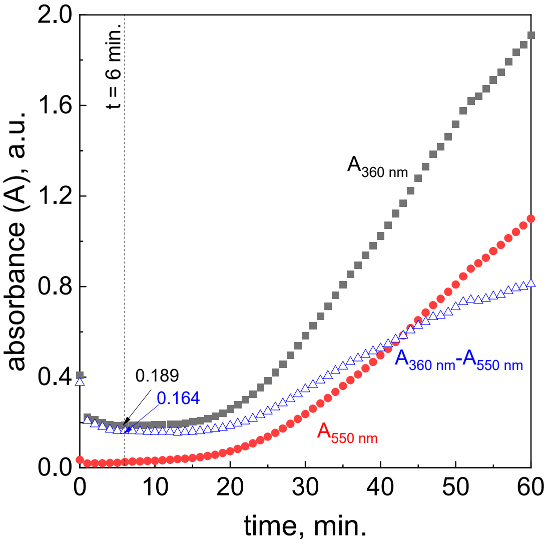

Figure S6. The sample of kinetic curves registered at two wavelength for process of Pt(IV) ion reduction using ascorbic acid and kinetic curve containing correction related to turbidity level ($A_{360\ nm}$-$A_{550\ nm}$). Conditions: $C_{0,Pt(IV)}$ = 1.0 mM, $C_{0,\ H2Asc}$ = 60 mM, T = 313 ± 0.1 K, I = 0.06 M, pH = 2.5.
